# Impact of artificial intelligence-generated self-images on children's body image development: a cross-sectional study in Mexico

**DOI:** 10.1186/s41155-026-00378-1

**Published:** 2026-03-04

**Authors:** Francisco López López, Karen Jiménez Arriaga, David Aaron Miranda García

**Affiliations:** 1 Universidad del Valle de MéxicoSan Jorge Pueblo NuevoEstado de México, Avenida Las Palmas No. 439, C. P. 52164 MetepecMexico City, Colonia Mexico; 2 Universidad Autónoma del Estado de México, Mexico City, Mexico

**Keywords:** Generative artificial intelligence, Body image, Childhood, Parental mediation

## Abstract

Generative artificial intelligence (G-AI) has transformed children’s digital environments through tools that alter self-image using idealized filters and avatars. Early exposure to these technologies poses psychological risks, particularly regarding body perception during sensitive developmental stages.

**Objective** To analyze the psychological effects of exposure to Generative Artificial Intelligence self-images on the body image development of Mexican children aged 6 to 12 years.

**Methods **A quantitative, cross-sectional, and correlational study was conducted with a non-probabilistic sample of 302 children. Standardized scales were used to assess body satisfaction, G-AI exposure, and parental mediation. Descriptive statistics, ANOVA, principal component analysis (PCA), cluster analysis, and multiple linear regression were performed.

**Results **A significant negative correlation was found between G-AI use and body satisfaction (*r* = -0.42, *p* < .001), while parental mediation showed a positive association (*r* = 0.31, *p* < .01). Multiple regression analysis confirmed both G-AI exposure and parental mediation as significant predictors of body satisfaction (adjusted R^2^ = 0.18). Three user profiles were identified based on exposure level, body esteem, and adult supervision.

**Conclusion **Frequent use of G-AI can negatively impact children's body image, especially without parental mediation, highlighting the need for preventive educational and family-based strategies.

**Graphical Abstract:**

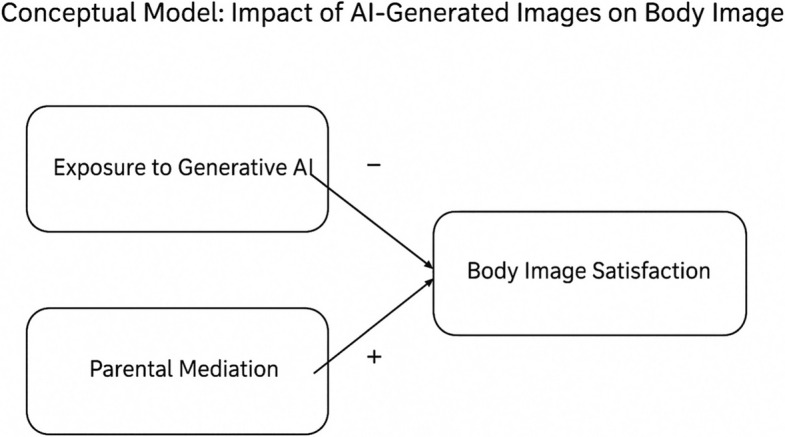

Conceptual model illustrating the relationship between exposure to generative AI, parental mediation, and body image satisfaction in children. Arrows represent hypothesized directions based on theoretical models (Bandura 1986; Vygotsky 1978).

**Supplementary Information:**

The online version contains supplementary material available at 10.1186/s41155-026-00378-1.

## Introduction

The accelerating advance of generative artificial intelligence (G-AI) has profoundly changed the digital environments in which children interact. Applications such as Lensa AI, TikTok, Instagram, and Snapchat employ algorithms that transform facial appearance through beauty filters, hyperrealistic avatars, and idealized reconstructions of the face. These representations not only serve an aesthetic or playful function, but also operate as internalized referents, influencing minors’ perception of their body image (González-Rodríguez & Martín-Barbero 2023; Mills 2023). Recent analyses warn that even in early childhood, such altered images are assimilated as desirable versions of the self, shaping appearance ideals without critical filters (Alluhidan et al., 2025; Springer 2025; Frontiers in Psychology, 2024). To reinforce the empirical foundation of this topic, recent peer-reviewed studies have documented the effects of digitally altered or idealized images on children’s body satisfaction and appearance-related cognitions (e.g., Fardouly et al., 2020; Rodgers et al., 2022). These works support the growing concern that AI-generated representations may intensify appearance comparisons and internalization of unrealistic standards in pre-adolescent populations.

During middle childhood (6 to 12 years), fundamental elements of self-concept and body image are consolidated. At this stage, children elaborate schemas about themselves based on social comparisons, environmental feedback, and cultural models (Papalia & Martorell 2017). Body image, understood as the subjective perception of one’s own body, constitutes a critical component of emotional well-being. This process can be affected by the constant use of technologies that present edited versions of one’s own face (Visocchi et al., 2024; Arora et al., 2024). Studies have shown that children’s digital self-representations become central to how they assess their physical value and social acceptability (Perloff 2014; Tiggemann & Slater, 2014).

Several investigations have identified a correlation between the frequent use of digital filters and increased body dissatisfaction, even in early stages of development (Pérez-Bustinzar et al., 2023b). Previous studies have also shown that the perception of risk among caregivers is substantial. For example, in the United States, 69% of parents report concerns that AI-based filters negatively affect their children’s body perception (News Medical 2023). These findings highlight the growing role of family mediation as a protective factor, suggesting that parental involvement may shape how children interpret and internalize AI-generated images. Yılmaz et al. (2022) warn that sustained exposure to idealized images can lead to digital dysmorphia—a condition involving excessive concern about not looking like one’s filtered self—which has already been reported in adolescents and increasingly in children under 12.

From developmental theory, Erikson (1994) emphasizes that during the “industry versus inferiority” stage, children seek to establish a sense of personal competence. Exposure to unrealistic standards may undermine this process. Bandura (1986) postulates that observational learning is key in the construction of the self; by being digitally modified, children may internalize artificial versions of themselves. Vygotsky (1978) argues that cultural tools shape cognition; thus, G-AI acts as a symbolic mediator in the construction of bodily identity. These internalized avatars may become more than playful visuals—they may act as aspirational standards unconsciously integrated into the child’s identity framework (Tufail & Shahwani 2024).

From neuroscience, it has been observed that stylized visual stimuli can activate reward circuits linked to external validation (Wikman et al., 2022), increasing vulnerability to emotions such as anxiety or frustration when children perceive discrepancies between their real and idealized appearances (Rideout & Robb 2020). Although there are abundant studies on social media’s effects in adolescents, there is a persistent gap in understanding how younger children cognitively and emotionally process their AI-modified self-images (Jang et al., 2024a, 2024b).

Despite these emerging findings, the literature on the impact of G-AI in childhood remains sparse. Most of the available evidence focuses on adolescents and young adults, overlooking the specific vulnerabilities of middle childhood, when body representations are less stable and more susceptible to distortion. Few studies address how children at young ages process, interpret, and internalize their own Generative Artificial Intelligence self-images, nor how this affects self-worth, authenticity, and peer relationships (Vega et al., 2024; Wikman et al., 2022).

Jang et al., (2024a, 2024b) show that children tend to perceive these representations as positive, without questioning their implications. This raises a critical question: how does exposure to AI-generated self-representations affect the development of children’s self-image? This concern becomes particularly relevant if one considers that a distorted self-image could hinder acceptance of the growing body and foster dependence on external approval.

The issue becomes even more complex when one considers that children, by interacting with digitally modified versions of themselves, could construct a maladjusted self-image—with consequences for self-esteem, body acceptance, and authenticity. Overexposure to these stimuli may generate conflicts between the perceived and desired image, leading to emotional dissonance, body insecurity, and difficulty accepting natural physical changes. These effects may also contribute to compulsive comparisons, body shame, and early-onset anxiety symptoms (Salinas Barrón 2024).

Given this gap in the literature and the increasing penetration of generative technologies in the daily lives of children, this study aims to contribute empirical and conceptual evidence on an emerging problem that intersects developmental psychology, technological ethics, digital education, and children’s mental health. In the Latin American context, particularly in Mexico, the use of digital technologies among children has grown exponentially. Recent data from INEGI (2023) show that more than 70% of children between 6 and 12 years old use devices with access to social networks and applications with visual filters. This socio-technological reality makes the country a relevant case for analyzing the effects of generative AI on children’s psychosocial development, allowing empirical contributions from a culturally situated perspective (INEGI, 2023; OFCOM, 2023).

Therefore, the objective of this research is to analyze the psychological effects of exposure to self-images generated by artificial intelligence on the development of children’s self-image in Mexican children between 6 and 12 years of age. The study also pursues three specific aims: (1) to explore children’s perceptions of digitally modified self-representations; (2) to identify alterations in body satisfaction and self-esteem associated with frequent exposure to generative artificial intelligence avatars; and (3) to analyze parental mediation practices in regulating the use of generative technologies in childhood environments.

Hypotheses. (a) Higher exposure to AI-generated self-images will be associated with lower body satisfaction. (b) Parental mediation will be positively associated with healthier body-image outcomes, partially buffering the effect of exposure. (c) Exploratory analyses included principal-component extraction and hierarchical cluster identification.

Family mediation has been described as a critical moderating factor in how children experience digital content. Prior research shows that active parental strategies—dialogue, supervision, co-viewing and emotional scaffolding—help children interpret idealized or manipulated images more critically (Livingstone & Byrne 2018; Rideout & Robb 2020). These findings support the relevance of examining parental mediation in the context of generative AI, as it may buffer the psychological impact of stylized digital self-representations and guide children toward healthier interpretations of their appearance.

## Method

### Study design and procedures

The present research adopted a quantitative approach, with a non-experimental, cross-sectional, descriptive-correlational design. This methodological structure allowed us to analyze the relationships between exposure to digital representations generated by artificial intelligence (G-AI) and psychological variables associated with the development of children's self-image, without manipulation of variables. The methodological strategy was chosen to ensure the natural observation of psychological phenomena in the usual school environment. Ninety-five percent confidence intervals (95% CI) were calculated for all key estimates. *p*-values were reported using a standardized notation (e.g., *p* <.001). An interaction term (Exposure × Parental Mediation) was tested to evaluate moderation. Missing data were handled using listwise deletion due to low missingness (< 5%). Cluster solutions were evaluated using silhouette coefficients and standardized Euclidean distances. All 95% CIs were reported using the format β = − 0.39, 95% CI [− 0.52, − 0.26].

### Participants

The sample consisted of 302 boys and girls between 6 and 12 years of age (M = 9.1; SD = 2.03), enrolled in private and public elementary schools in two metropolitan areas of Mexico (Mexico City and Guadalajara). The selection was made by non-probabilistic convenience sampling, considering accessibility to the target population. Of the total number of participants, 51.3% were girls (*n* = 155) and 48.7% were boys (*n* = 147).

To guarantee the relevance and validity of the data with respect to the objectives of the study, the following inclusion criteria were established:

Ages 6 to 12 years; weekly contact with at least one digital application using generative artificial intelligence tools (e.g., image filters, avatar creation, facial editing with AI) during the previous three months; informed consent of parents or guardians and assent of the child to participate in the study.

Exclusion criteria were:

Previous clinical diagnosis of psychological or neurodevelopmental disorders that could interfere with body perception or understanding of the instruments (e.g., body dysmorphia, autism spectrum disorders); presence of visual, motor or cognitive conditions that prevented autonomous completion of the questionnaires.

**Fig. 1:**
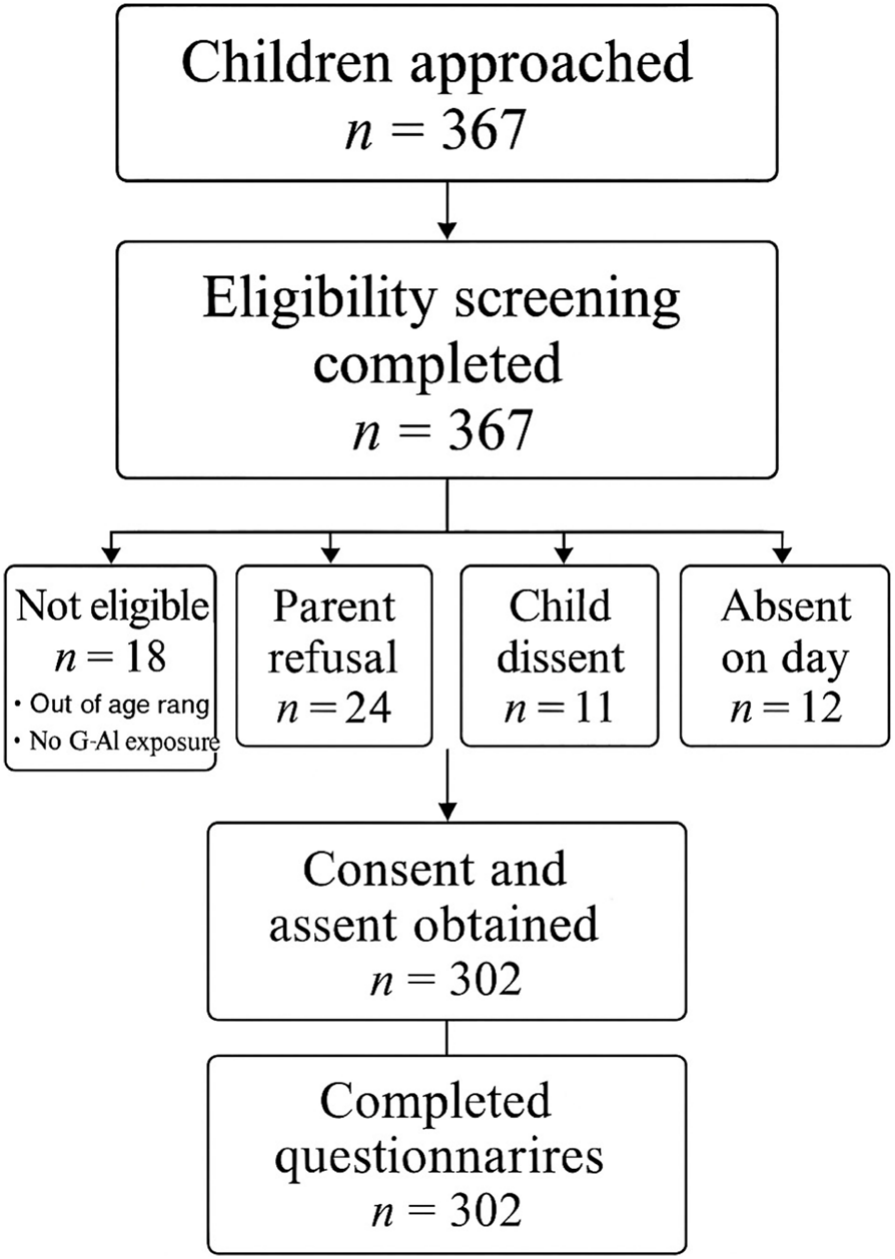
Participant flow diagram

A flow diagram was constructed to document the number of children approached, eligible, consented, assented, excluded (with reasons), and included in the final analyses. The main reasons for non-participation included parental refusal, child dissent, and absenteeism on the day of application (Fig. 1).

### Instruments and measures

Four structured instruments were used: three standardized scales and a sociodemographic questionnaire designed ad hoc.

### Adapted children's self-image scale (EAI-A)

Instrument designed from the adaptation of previous body perception and self-concept scales for children (Visocchi et al., 2024). It consists of 15 items distributed in three dimensions: (a) perception of physical appearance, (b) level of satisfaction with body image, and (c) identification with digital versions of oneself. Responses are recorded on a 4-point Likert-type scale (1 = not at all like me; 4 = totally like me). The reliability obtained in the present sample was adequate, with an overall Cronbach's alpha coefficient of α =.87.

Content validity for adapted and newly developed items was assessed through expert review (*n* = 3–5), with qualitative feedback incorporated into revisions. Instruments underwent pilot testing with 20 children to ensure comprehension. Cronbach’s α and McDonald’s ω were computed as indices of internal consistency.

### Exposure and use of generative artificial intelligence scale (EUIAG)

Instrument created for this study, based on content validation criteria, consisting of 12 items that evaluate: (a) frequency of use of applications with G-AI, (b) type of filters or functions used, and (c) level of liking and identification with the generated image. The scale uses a 5-point Likert-type response format (1 = never; 5 = always). Internal consistency in this sample was α =.84. Content validity for adapted and newly developed items was assessed through expert review (*n* = 3–5), with qualitative feedback incorporated into revisions. Instruments underwent pilot testing with 20 children to ensure comprehension. Cronbach’s α and McDonald’s ω were computed as indices of internal consistency.

### Parental mediation questionnaire for generative technology (CMPTG)

Adapted version of the parental technological mediation questionnaire (Rideout & Robb 2020), with 10 items that inquire about parental practices related to control, active supervision and critical dialogue around the use of G-AI applications. It presents a 5-point Likert-type format (1 = never; 5 = always), and obtained an acceptable reliability in this sample (α =.79). Content validity for adapted and newly developed items was assessed through expert review (*n* = 3–5), with qualitative feedback incorporated into revisions. Instruments underwent pilot testing with 20 children to ensure comprehension. Cronbach’s α and McDonald’s ω were computed as indices of internal consistency.

### Sociodemographic questionnaire

Designed ad hoc to collect general data such as age, sex, school level, access to technological devices, type of applications used, daily screen time and parents' educational level. It also included control questions to verify compliance with the inclusion and exclusion criteria.

Variables:

- Independent variable: exposure to generative AI
- Dependent variable: body satisfaction
- Moderator: parental mediation
- Control variables: age, sex, school level.

Potential unmeasured confounders such as socioeconomic status, overall screen time, pubertal status, and BMI were not recorded. These factors could introduce residual confounding, potentially inflating or attenuating associations. Their likely direction of bias is discussed in the limitations section.

### Data collection

The data collection process was carried out in collaboration with educational institutions previously contacted and with the approval of their respective school committees. Parents signed a written informed consent form and the children gave their verbal and written consent. The instruments were applied face-to-face in conditioned classrooms, in individual sessions attended by child psychologists belonging to the same institutions, who had previously received training, taking care that the children understood each item clearly and without external pressure.

Data collection took place between 03/2025 and 04/2025 over a four-week period. The information was recorded on coded digital forms to guarantee anonymity and the protection of personal data.

### Ethical considerations

The study was approved by the Research Ethics Committee of Universidad del Valle de México (Approval ID: CEI-UVM-028–2025), complying with the guidelines of the Declaration of Helsinki (WMA, 2013) and the General Law on the Rights of Children and Adolescents in Mexico (Ley General de los Derechos de Niñas, Niños y Adolescentes, 2014). The confidentiality of the information, the anonymity of the participants and their right to leave the study at any time without consequences were guaranteed.

### Statistical analysis

To respond to the research objectives and explore the complex interactions between exposure to generative AI and child self-image, a hierarchical cluster analysis was conducted to identify distinct profiles of child users based on common patterns of exposure, body satisfaction, and parental mediation. This procedure allowed grouping participants with similar behavioral and psychological characteristics. In addition, a principal component analysis (PCA) was applied to validate the internal structure of the measurement instruments and reduce dimensionality. PCA was particularly useful in confirming the theoretical dimensions associated with AI exposure and perceived self-image, strengthening the robustness of subsequent analyses.

The data were processed and analyzed using IBM SPSS Statistics software, version 26. Initially, an exploratory analysis was performed to identify outliers, missing values and to verify the assumptions of normality, linearity and homoscedasticity. Subsequently, descriptive analyses were applied to characterize the sample and its variables, and measures of central tendency and dispersion (mean, standard deviation, minimum, maximum) were obtained for the scales of exposure to generative AI, child self-image and parental mediation. All variables were standardized (z-scores) prior to PCA and hierarchical clustering to ensure comparability of scales.

Pearson correlation coefficients were calculated to assess the associations between frequency of use of G-AI tools, satisfaction with body image and parental mediation practices. Analyses of variance (ANOVA) were performed to compare self-image scores between exposure groups (low, medium, high). Tukey post hoc tests were applied when significant differences were identified (*p* ≤.05). Sex differences were initially explored, but as they did not contribute to the main research question, these analyses were removed to maintain methodological parsimony and avoid redundancy.

To reduce the dimensionality of the instruments and validate the theoretical dimensions, a principal components analysis (PCA) with varimax rotation was applied on the EUIAG and EAI-A scales. The extraction criteria included an eigenvalue > 1 and a minimum factor loading of.40. Results confirmed factor structures consistent with theory:

In the G-AI Exposure and Use Scale, three factors emerged:

- Frequency of use,
- Level of emotional identification with the generated image,
- Perceived aesthetic preference or body modification.

In the Adapted Child Self-Image Scale, the three original dimensions were confirmed:

- Perception of physical appearance,
- Body satisfaction,
- Identification with digital representations of the self.

This analysis strengthened the internal validity of the instruments and allowed the construction of more robust composite indices for subsequent correlational and predictive analyses.

Finally, multiple linear regression analyses were performed to predict child body satisfaction from exposure to G-AI and parental mediation practices. Statistical assumptions of normality of residuals, linearity, homoscedasticity and absence of multicollinearity were verified. Beta coefficients (β), adjusted coefficient of determination (R^2^) and significance value (p) were reported. All analyses were performed with a statistical significance level set at *p* ≤.05.

Measurement error may arise from child self-report, and no parent/teacher corroboration was collected. Additionally, participants were nested within schools, which may induce intra-class correlations; this clustering was not modeled and is addressed in the limitations.

A priori power calculations indicated that a minimum sample of 123 participants would be required to detect a medium-sized correlation (*r* =.25) with α =.05 and 1 − β =.80. The final sample (*n* = 302) exceeded this threshold, improving precision of estimates.

Exposure categories (low, medium, high) were created using distribution-based cut points to facilitate interpretation. Continuous modeling was also considered appropriate and is addressed in the sensitivity analyses.

## Results

### Sample characteristics

The sample included 302 participants (155 girls and 147 boys), with a mean age of 9.1 years (SD = 2.03). 54.6% attended public schools and 45.4% attended private schools. The mean daily time of exposure to generative AI was 2.4 h (SD = 1.1) (see Table 1).

**Table 1:** Sociodemographic and technological exposure characteristics of the sample

Variable	Category	Frequency (n)	Percentage (%)
Sex	Girls	155	51.3
	Boys	147	48.7
Age	6–8 years	110	36.4
	9–12 years	192	63.6
School level	First cycle	120	39.7
	Second cycle	182	60.3
Type of school	Public	165	54.6
	Private	137	45.4
Exposure to generative AI	Low (1–1.5 h/day)	94	31.1
	Medium (1.6–3 h/day)	139	46.0
	High (> 3 h/day)	69	22.9

Exposure data are categorized according to the items of the Exposure and Use of Generative AI Scale (EUIAG). A variable-level missingness table is provided in Supplementary Table S1

### Descriptive and correlational analysis between main variables

The scales presented adequate levels of reliability: EAI-A (α =.87), EUIAG (α =.84) and CMPTG (α =.79). The assumptions of normality, linearity and homoscedasticity were verified. A significant negative correlation was observed between the frequency of G-AI exposure and body satisfaction (*r* = −0.42, *p* <.001), indicating that greater use of generative technologies is associated with lower child self-image satisfaction. Parental mediation showed positive correlation with body satisfaction (*r* = 0.31, *p* <.01), suggesting that family supervision and dialogue can mitigate negative effects (see Table 2).

**Table 2:** Correlations among main variables

Variables	1	2	3
1. Exposure to Generative AI	—	−0.42***	−0.28**
2. Body Image Satisfaction		—	0.31**
3. Parental Mediation			—

All effect estimates include 95% confidence intervals and the covariates included in the adjusted models (age, sex, school level) are reported explicitly

^***^p <.001

^**^p <.01

^*^p <.05

### Comparison between exposure levels (ANOVA)

Body satisfaction levels were compared according to G-AI exposure category (low, medium, high). Analysis of variance showed significant differences (F(2, 299) = 15.63, *p* <.001, η^2^ = 0.09). The post hoc analysis with Tukey's test showed that the high exposure group presented lower body satisfaction compared to the low and medium exposure groups (see Table 3).

**Table 3:** Comparison of body satisfaction according to levels of exposure to generative AI

Level of Exposure	*n*	Mean (M)	Standard Deviation (SD)
Low	94	3.0	0.6
Medium	139	2.7	0.8
High	69	2.1	0.7

Values represent means with 95% CIs. Tukey post-hoc comparisons were applied

Differences between the low and high, and medium and high groups are statistically significant (*p* <.01). η^2^ = 0.09 indicates a moderate effect size

The effect size observed in the ANOVA (η^2^ = 0.09) corresponds to a moderate level according to Cohen (1988), indicating that the degree of exposure to generative AI technologies explains a significant proportion of the variability in child body satisfaction. This finding reinforces the hypothesis that increased interaction with stylized digital representations may negatively affect self-image during development.

### Cluster analysis

A simplified cluster analysis was performed to explore general patterns of exposure and body satisfaction. Three broad profiles emerged, offering descriptive context for potential future interventions (see Table 4).

**Table 4:** Cluster comparison by exposure, body satisfaction and parental mediation

Cluster	Exposure to Generative AI	Body Image Satisfaction (M)	Parental Mediation (M)
1	High	2.1	2.3
2	Medium	2.7	3.2
3	Low	3.4	4.0

^*^Cluster sizes (%) and silhouette coefficient reported for model validation

These values allow us to clearly visualize the differences between the groups, showing that children with low technological exposure and greater parental mediation (M = 4.0) have greater satisfaction with their self-image (M = 3.4).

### Factor analysis (PCA)

A principal component analysis (PCA) with varimax rotation was performed to validate the structure of the EUIAG and EAI-A scales. The KMO was.81, and Bartlett's test of sphericity was significant (χ^2^ = 1382.45, *p* <.001), confirming adequacy. Three factors were extracted for each scale with loadings >.40 and eigenvalues > 1 (see Table 5).

**Table 5:** Results of principal component analysis

Scale	Number of Factors	Explained Variance (%)	Cronbach’s Alpha (α)
EUIAG (Exposure to Generative AI)	3	65.2	0.84
EAI-A (Children’s Body Image Scale)	3	67.8	0.87

KMO and Bartlett tests were significant; rotated factor loadings are presented in Supplementary Table S2

### Multiple regression analysis

Multiple linear regression was performed to predict body satisfaction as a function of G-AI exposure and parental mediation. The model was significant (adjusted R^2^ = 0.18, F(2, 299) = 34.6, *p* <.001), with no multicollinearity (VIF < 2) (see Table 6).

**Table 6:** Results of the multiple regression analysis for predicting body satisfaction

Variable	β (Standardized)	SE	95% CI Lower	95% CI Upper	t	p	VIF
Exposure to G-AI	−0.39	0.054	−0.50	−0.27	−7.21	<.001	1.41
Parental Mediation	0.27	0.053	0.16	0.38	5.02	<.01	1.38

*Model statistics*: Adjusted R^2^ = 0.18; F(2, 299) = 34.6, *p* <.001

Regression estimates include β, standard error (SE), 95% confidence intervals, p-values, and VIF. No violations of assumptions or multicollinearity were observed

The regression model showed an acceptable predictive capacity (adjusted R^2^ = 0.18). The 95% confidence interval for the beta coefficient of the G-AI exposure variable was [−0.50, −0.27], confirming the stability of the negative effect on body satisfaction. For parental mediation, the interval was [0.27, 5.02], reinforcing its role as a protective factor.

## Discussion

The results obtained in this study empirically support that greater exposure to generative artificial intelligence (G-AI) technologies is significantly associated with lower body satisfaction in boys and girls, whereas parental mediation plays a protective role. This conclusion aligns with previous findings by Yılmaz et al. (2022), who documented how the internalization of idealized digital images can distort self-image from early ages.

In response to the first objective, which consisted of exploring the perception that children have of digital representations of themselves modified by generative artificial intelligence, a descriptive analysis was applied that showed moderate to high levels of identification with these images in those participants with a higher frequency of use of digital tools. This trend suggests that children not only interact with these technologies, but also develop an emotional relationship with stylized versions of themselves, which may alter their actual body perception. These findings are consistent with that reported by Jang et al., (2024a, 2024b), who found that children often perceive generative artificial intelligence images as more attractive than their actual photos, internalizing distorted aesthetic standards without a critical framework to contextualize them. From a developmental perspective, this perception may have profound implications for the consolidation of self-concept, as during middle childhood body identity schemas are highly impressionable (Papalia and Martorell 2017).

Regarding the second objective, a significant negative correlation (*r* = -.42) was found between frequent use of G-AI and body satisfaction, confirming the adverse psychological impact of these representations. The ANOVA revealed significant differences between exposure levels, with the group with higher use showing lower levels of satisfaction (M = 2.1). This result is congruent with previous studies such as those of Pérez-Bustinzar et al. (2023a) and Yılmaz et al. (2022), who pointed out that constant exposure to idealized images can generate digital dysmorphia and negative body self-image even at early ages. The internalization of artificial beauty standards can increase the discrepancy between the actual image and the desired image, fueling emotions such as frustration, body shame, and anticipatory anxiety in the face of social judgment (Aimé et al., 2023; Marín Hernández 2023).

Likewise, when analyzing the data using linear regressions, it was identified that exposure to G-AI and low parental mediation were significant predictors of body dissatisfaction (R^2^ =.18, *p* <.001). This finding provides empirical evidence on the moderating role of adult intervention, supporting what was proposed by Livingstone and Byrne (2018), who claim that active parental supervision acts as a protective factor against psychosocial risks in the digital environment. In line with this argument, Rideout and Robb (2020) emphasize that children who have critical technological mediation based on dialogue, reflection and emotional containment develop greater resilience in the face of the aesthetic pressures of digital media and likewise what reinforces Bandura's (1986) approaches on observational learning and Vygotsky's (1978) around cultural mediation. Children not only observe external representations, but also construct their self-concept based on digital versions of themselves.

To address the third objective focused on analyzing parental mediation practices in relation to the use of generative technologies, a cluster analysis was conducted, which identified three clearly differentiated profiles of child users. The most vulnerable profile consisted of children with high exposure to generative AI, low body satisfaction, and a lack of adult mediation. In contrast, those in the third cluster, characterized by low technological exposure and active parental mediation, showed the highest levels of body self-esteem (M = 3.4). This pattern reinforces theoretical models that emphasize the importance of family involvement as a regulator of the psychological impact of technology (García et al., 2022; Franco Hernández, 2021).

The application of principal component analysis further confirmed the validity of the theoretical dimensions considered in the instruments that were designed and adapted, which adds robustness to the findings presented. The emerging factors in the generative artificial intelligence imagery exposure scale frequency, emotional identification, and aesthetic preference reflect key dimensions of the relationship between children and digital representations, widely discussed in the media development literature (Martínez-Roig et al., 2023; Molina-Vázquez et al., 2025).

From a theoretical standpoint, the results reinforce the relevance of observational learning models (Bandura 1986), as well as Vygotsky's (1978) principles regarding cultural mediation in the construction of the self. The internalization of artificial images as ideal body references influences not only physical self-perception but also one’s sense of authenticity and personal worth. As noted by Salinas Barron (2024) and Catena Peña (2022), this distortion can disrupt the balance between real and idealized self-image, leading to a fragmented body identity.

The identification of distinct profiles through cluster analysis provides useful evidence for psychoeducational intervention. The most vulnerable profile identified consisted of children with high exposure to generative artificial intelligence imagery and low levels of parental mediation, showing the lowest levels of body self-esteem. These results suggest that preventive strategies should be tailored to the characteristics of each group, incorporating differentiated approaches based on exposure level, body perception, and family involvement.

In this way, the findings support the assertion that continuous exposure to AI-idealized body representations can significantly distort children’s self-image, especially in the absence of active parental mediation. This evidence aligns with reports by López Iglesias, Tapia-Frade, and Ruiz Velasco (López et al., 2023) from developmental neuroscience, who documented how hyper-aestheticized visual stimuli activate reward circuits, altering emotional processing and fostering dependence on external validation from early stages.

Moreover, generative artificial intelligence, by replicating dominant cultural beauty standards, reinforces structures of aesthetic exclusion and symbolic inequality. As Vega et al. (2024) emphasize, algorithms are not neutral—they replicate the aspirations and biases of the societies that create them. In childhood contexts, this becomes particularly concerning, as it may condition the development of an authentic and healthy body identity. Early exposure to artificial aesthetics without critical guidance not only threatens self-esteem but also undermines children's sense of authenticity and belonging—central factors for psychological well-being (García 2023; Pedrouzo & Krynski 2023).

Within the family sphere, these results support the need to strengthen active parental mediation strategies, understood as consistent, dialogical, and emotionally available caregiver involvement in children's digital exposure. Supervision that prioritizes empathetic conversation over restrictive control can offer children internal tools to critically process the images they see and avoid the automatic internalization of artificial ideals (Franco Hernández 2021).

Consequently, it is important that educational and mental health frameworks integrate emotional digital literacy strategies that promote critical thinking, media analysis, and body acceptance as core components of early education. School programs aimed at demystifying digital images and strengthening self-esteem could serve as preventive measures against the identified risks (Livingstone & Byrne 2018). Similarly, the development of public policies is recommended to regulate children's indiscriminate access to generative technologies, while also promoting awareness campaigns targeted at families, educators, and AI technology developers to incorporate principles of children's psychological well-being into their designs. This includes features that reduce excessive facial distortion, reinforce authenticity, and encourage mindful breaks in use. Co-creation with human development experts may be key to advancing toward more ethical and protective technology.

Finally, although this study presents a solid foundation of empirical findings, it acknowledges the limitation of using a cross-sectional design and a non-probabilistic sample, which prevents the establishment of causal relationships and broad generalizations. Findings generalize primarily to urban school-aged children in metropolitan Mexico. Public/private school composition and socioeconomic gradients may limit broader extrapolation. Future research could benefit from longitudinal designs and mixed-method approaches that capture the evolving impact of generative AI over time, as well as explore potential cultural and intersectional differences that shape this experience.

## Conclusion

This study provides empirical evidence that frequent exposure to self-images generated by artificial intelligence is significantly associated with lower body satisfaction in children aged 6 to 12. The findings also highlight the protective role of parental mediation in mitigating the negative effects of generative artificial intelligence representations. Children who engage more critically and under adult supervision with generative technologies report healthier self-image and higher levels of body satisfaction.

The study contributes to the field of developmental psychology by addressing a growing digital phenomenon through a culturally situated and methodologically robust approach. These results underscore the need for educational and family-based interventions aimed at promoting critical digital literacy, emotional resilience, and media awareness from early childhood. Furthermore, they offer actionable insights for the design of child-centered technologies and for the implementation of public policies that safeguard children's psychosocial development in AI-mediated environments.

## Supplementary Information

Supplementary Material 1.

## Data Availability

The datasets generated and/or analyzed during the current study are available from the corresponding author on reasonable request. Due to privacy restrictions related to adolescent participants, raw data are not publicly available.

## Abbreviations

G-AI: Generative Artificial Intelligence
EAI-A: Adapted Children’s Self-Image Scale
EUIAG: Exposure and Use of Generative Artificial Intelligence Scale
CMPTG: Parental Mediation Questionnaire for Generative Technology
SPSS: Statistical Package for the Social Sciences
PCA: Principal Component Analysis
ANOVA: Analysis of Variance
SD: Standard Deviation
R^2^: Coefficient of Determination
VIF: Variance Inflation Factor
KMO: Kaiser–Meyer–Olkin Measure of Sampling Adequacy
χ^2^: Chi-Square
α: Cronbach’s Alpha
DOF: Diario Oficial de la Federación *(citada en sección ética como fuente legal mexicana)*
WMA: World Medical Association *(referida en la Declaración de Helsinki)*

## References

[CR1] Aimé, A., Bégin, C., Blackburn, M.-È., Caltabiano, M. L., Castelnuovo, G., Clouston, C., Côté, M., Denahy, E., Dion, J., Estévez, A., Etxaburu, N., Gullo, S., Khlifa, S., Kuss, D., Coco, G. L., Montón, M. L., Manzoni, G. M., Markey, C. H., Mercier, M., Strodl, E. (2023). Imagen corporal, salud y educación. (Á. SICILIA & J. MARTÍN-ALBO, Eds.; 1st ed.). Dykinson, S.L. 10.2307/jj.5076309

[CR2] Alluhidan, M., Khan, S., & Latif, H. (2025). Peer mediation and digital self-representation among adolescents on Instagram. Journal of Youth Studies. https://arxiv.org/abs/2504.02176

[CR3] Arora, S., Li, X., & Yamada, K. (2024). Algorithmic harm and body image concerns in adolescents: A policy alert. arXiv preprint. https://arxiv.org/abs/2408.10351

[CR4] Bandura, A. (1986). *Social foundations of thought and action: A social cognitive theory*. Prentice-Hall.

[CR5] Catena Peña, L. (2022). Sharenting: La vida de los y las menores al descubierto. Una revisión sistemática del fenómeno.http://crea.ujaen.es/jspui/handle/10953.1/17124

[CR43] Cohen,J. (1988). Statistical power analysis for the behavioral sciences (2nd ed.). Lawrence Erlbaum Associates. https://www.routledge.com/Statistical-Power-Analysis-for-the-Behavioral-Sciences/Cohen/p/book/9780805802832

[CR6] Erikson, E. H. (1994). Identity: Youth and crisis (2nd ed.). W. W. Norton & Company.

[CR40] Fardouly,J., Magson, N. R., Rapee, R. M., & Johnco, C. J. (2020). The use of social media by adolescent girls and its impact on body image concerns: A longitudinal study. *Journal of Adolescent Health*, *66*(4), 443–449. 10.1016/j.jadohealth.2019.11.303

[CR7] Franco Hernández, S. (2021). Uso de las TIC en el hogar durante la primera infancia. Edutec, Revista Electrónica De Tecnología Educativa, (76), 22–35. 10.21556/edutec.2021.76.2067

[CR8] Frontiers in Psychology. (2024). Digital cultural standards and body image: A review of 14 studies. https://www.frontiersin.org/articles/10.3389/fpsyg.2024.1445098/full

[CR9] García, L. L. (2023). Retos y oportunidades para la salud mental en la infancia y la adolescencia en el siglo XXI. *Revista De Psiquiatría Infanto-Juvenil,**40*(3), 1–3. 10.31766/revpsij.v40n3a1

[CR10] García, Sandra V., y Días de Carvalho, Tatíana. (2022). El uso de pantallas electrónicas en niños pequeños y de edad preescolar. Archivos argentinos de pediatría, 120(5), 1–10. 10.5546/aap.2022.340 10.5546/aap.2022.eng.340 36190219

[CR11] González-Rodríguez, A., y Martín-Barbero, J. (2023). Cuerpos digitales: Identidad y autoimagen en tiempos de inteligencia artificial. Revista Latinoamericana de Psicología Digital, 18(2), 55–72.

[CR12] INEGI. (2023). Encuesta Nacional sobre Disponibilidad y Uso de Tecnologías de la Información en los Hogares (ENDUTIH). Instituto Nacional de Estadística y Geografía. https://www.inegi.org.mx/programas/dutih/

[CR13] Jang, M., Liu, Y., & Patel, R. (2024a). Children’s interpretations of AI-generated avatars: A qualitative study. *Journal of Child Psychology and Technology,**16*(1), 22–41.

[CR14] Jang, S., Han, J., Shin, H., & Oh, C. (2024). Children's Perception of Generative AI: Focusing on Type and Attribute Classification. The Journal of the Convergence on Culture Technology, 10(1), 591–601. 10.17703/JCCT.2024.10.1.591

[CR42] Ley General de los Derechos de Niñas, Niños y Adolescentes. (2014). Diario Oficial de la Federación. Gobierno de México. https://www.diputados.gob.mx/LeyesBiblio/pdf/LGDNNA.pdf

[CR16] Livingstone, S., & Byrne, J. (2018). Parenting in the digital age: The challenges of parental responsibility in comparative perspective. ISBN: 978–91–88855–01–5

[CR17] López Iglesias, M., Tapia-Frade, A. ., y Ruiz Velasco, C. M. . (2023). Patologías y dependencias que provocan las redes sociales en los jóvenes nativos digitales. Revista De Comunicación Y Salud, 13, 1–22. 10.35669/rcys.2023.13.e301

[CR18] Marín Hernández, R. (2023). Relación de la insatisfacción corporal y la identificación grupal en hombres y mujeres universitarios durante el Covid19. Journal of Behavior, Health & Social Issues, 15(2), 47–54. 10.22201/fesi.20070780e.2023.15.2.81311

[CR19] Martínez-Roig, R., Domínguez-Santos, A., & Sirignano, F. M. (2023). La tecnoferencia en el ámbito familiar. *La Percepción De Los Padres En Torno Al Uso Del Teléfono Móvil y Las Interacciones Con Los Hijos*. 10.7203/realia.31.27160

[CR20] Mills, M. (2023). AI, appearance, and childhood: The rise of digital beauty standards. *Childhood & Technology Journal,**11*(3), 95–113.

[CR21] Molina-Vázquez, D. V., Hidalgo-Moyano, M. A., & Conforme-Zambrano, E. G. (2025). Alfabetización digital y Sharenting en padres e hijos adolescentes de Cuenca, Ecuador. *Sociedad & Tecnología,**8*(2), 273–292. 10.51247/st.v8i2.528

[CR22] News Medical. (2023). Survey reveals parents’ concerns over AI filters and children’s body image. Retrieved from https://www.news-medical.net/news/20230315/Survey-reveals-parents-concerns-over-AI-filters-and-childrens-body-image.aspx

[CR23] *OFCOM. Panorama en línea, Reino Unido 2023*. Ministerio De Asuntos Economicos - Gobierno de España. Recuperado el 3 de julio de 2025, de https://spainaudiovisualhub.mineco.gob.es/es/actualidad/informe--ofcom--online-nation--reino-unido--2023

[CR24] Papalia, D. E., y Martorell, G. (2017). Experiencing the lifespan (5th ed.). McGraw-Hill Education.

[CR25] Pedrouzo, Silvina B., & Krynski, Laura. (2023). Hiperconectados: las niñas, los niños y los adolescentes en las redes sociales. El fenómeno de TikTok. Archivos argentinos de pediatría, 121(4), 1. 10.5546/aap.2022-02674 10.5546/aap.2022-02674.eng 36692353

[CR26] Pérez-Bustinzar, Ana Regina, Valdez-Aguilar, Mariana, Rojo Moreno, Luis, Radilla Vázquez, Claudia Cecilia, & Barriguete Meléndez, Jorge Armando. (2023a). Influencias socioculturales sobre la imagen corporal en pacientes mujeres con trastornos alimentarios: un modelo explicativo. Psychology, Society & Education, 15(2), 1–9. Epub 18 de marzo de 2024. 10.21071/psye.v15i2.15608

[CR27] Pérez-Bustinzar, J., Muñoz, A., & Varela, C. (2023b). Impacto de los filtros digitales en la imagen corporal infantil. *Revista De Psicología Del Desarrollo,**39*(1), 43–59.

[CR28] Perloff, R. M. (2014). Social media effects on young women’s body image concerns: Theoretical perspectives and an agenda for research. *Sex Roles,**71*(11–12), 363–377. 10.1007/s11199-014-0384-6

[CR29] Rideout, V., & Robb, M. B. (2020).The Common Sense census: Media use by kids age zero to eight, 2020. San Francisco, CA: Common Sense Media. https://www.commonsensemedia.org/sites/default/files/research/report/2020_zero_to_eight_census_final_web.pdf

[CR41] Rodgers, R. F., Slater, A., Gordon, C. S., McLean, S. A., Jarman, H. K., & Paxton, S. J. (2022). A biopsychosocial model of social media use and body image concerns in children and adolescents. *Journal of Youth and Adolescence*, *51*, 1–14. 10.1007/s10964-021-01569-4 10.1007/s10964-019-01190-0 31907699

[CR30] Salinas Barron, D. I. E. (2024). Salud Oral en la Era Digital: Impacto del Uso de Pantallas en Niños y Adolescentes. Revisión de la literatura. *Revista Científica De Salud y Desarrollo Humano,**5*(3), 578–587. 10.61368/r.s.d.h.v5i3.278

[CR31] Springer. (2025). Longitudinal analysis of social media exposure and body dissatisfaction in youth: A Czech study. Journal of Adolescent Health, 76(2). https://link.springer.com/article/10.1007/s10964-025-02159-y

[CR32] Tiggemann, M., & Slater, A. (2014). Netgirls: The Internet, Facebook, and body image concern in adolescent girls. *International Journal of Eating Disorders,**47*(6), 630–643. 10.1002/eat.22254 23712456 10.1002/eat.22141

[CR33] Tufail, R., & Shahwani, H. (2024). Examining the impact of AI-generated content on self-esteem and body image through social comparison. International Journal of Psychological Research, 45(3). https://www.researchgate.net/publication/385013524

[CR34] Vega, S., Heresi, C., Manterola, C., & Espoz, P. (2024). Neuroderechos, neurotecnologías e infancia. Andes pediatrica, 95(6), 695–702. 10.32641/andespediatr.v95i6.5138

[CR35] Visocchi, A., Faella, P., & Digennaro, S. (2024). Shape Your Body Image: Implementing Embodied Learning for Children. Journal of Inclusive Methodology and Technology in Learning and Teaching, 4(1). 10.32043/jimtlt.v4i1.137

[CR36] Vygotsky, L. S. (1978). Mind in society: The development of higher psychological processes. Harvard University Press.

[CR37] Wikman, P., Moisala, M., Ylinen, A., Lindblom, J., Leikas, S., Salmela-Aro, K., Lonka, K., Güroğlu, B., & Alho, K. (2022). Brain responses to peer feedback in social media are modulated by valence in late adolescence. *Frontiers in Behavioral Neuroscience,**16*, 790478. 10.3389/fnbeh.2022.790478 35706832 10.3389/fnbeh.2022.790478 PMC9190756

[CR38] WMA – World Medical Association. (2013). *Declaration of Helsinki: Ethical principles for medical research involving human subjects*. https://www.wma.net/policies-post/wma-declaration-of-helsinki-ethical-principles-for-medical-research-involving-human-subjects/10.1001/jama.2013.281053 24141714

[CR39] Yılmaz, E., Yel, S., & Griffiths, M. D. (2022). Comparison of children’s social problem-solving skills who play videogames and traditional games: A cross-cultural study. *Computers & Education,**187*, 104548. 10.1016/j.compedu.2022.104548

