# Supplementary material for: Impact of artificial intelligence-generated self-images on children's body image development: a cross-sectional study in Mexico
Source: Psicol Reflex Crit. 2026 Mar 4;39:12. doi: 10.1186/s41155-026-00378-1 (PMC13018490; doi:10.1186/s41155-026-00378-1)
Supplement: Supplementary file 1 — Supplementary Material 1. [file 41155_2026_378_MOESM1_ESM.docx]

**Appendix A. Expanded Results from Cluster Analysis**

In addition to the PCA and regression analyses presented in the main results, a cluster analysis was conducted to explore distinct profiles of children based on their exposure to AI-generated images and body satisfaction scores. Three primary clusters emerged:
- Cluster 1: High exposure and low satisfaction (41%)
- Cluster 2: Moderate exposure and neutral satisfaction (33%)
- Cluster 3: Low exposure and high satisfaction (26%)

These groupings provide further insight into how different usage patterns relate to body image outcomes and can be used to design tailored intervention strategies.

**Supplementary Table S1. Missingness by variable**

| **Variable** | **n Missing** | **% Missing** |
| --- | --- | --- |
| Age | 0 | 0% |
| Sex | 0 | 0% |
| School Level | 0 | 0% |
| Exposure to G-AI (EUIAG total score) | 2 | 0.7% |
| Body Satisfaction (EAI-A total score) | 1 | 0.3% |
| Parental Mediation (CMPTG total score) | 2 | 0.7% |
| Daily Screen Time | 3 | 1.0% |
| Type of School | 0 | 0% |

**Note.** Missingness was <5% across all variables; therefore, listwise deletion was applied for correlational and regression analyses.
